# Role of Short Chain Fatty Acids and Apolipoproteins in the Regulation of Eosinophilia-Associated Diseases

**DOI:** 10.3390/ijms22094377

**Published:** 2021-04-22

**Authors:** Eva Maria Sturm, Eva Knuplez, Gunther Marsche

**Affiliations:** Otto Loewi Research Center, Division of Pharmacology, Medical University of Graz, 8010 Graz, Austria; eva.knuplez@medunigraz.at (E.K.); gunther.marsche@medunigraz.at (G.M.)

**Keywords:** eosinophils, eosinophilic disorders, lipids, apolipoproteins, HDL, short chain fatty acids

## Abstract

Eosinophils are key components of our host defense and potent effectors in allergic and inflammatory diseases. Once recruited to the inflammatory site, eosinophils release their cytotoxic granule proteins as well as cytokines and lipid mediators, contributing to parasite clearance but also to exacerbation of inflammation and tissue damage. However, eosinophils have recently been shown to play an important homeostatic role in different tissues under steady state. Despite the tremendous progress in the treatment of eosinophilic disorders with the implementation of biologics, there is an unmet need for novel therapies that specifically target the cytotoxic effector functions of eosinophils without completely depleting this multifunctional immune cell type. Recent studies have uncovered several endogenous molecules that decrease eosinophil migration and activation. These include short chain fatty acids (SCFAs) such as butyrate, which are produced in large quantities in the gastrointestinal tract by commensal bacteria and enter the systemic circulation. In addition, high-density lipoprotein-associated anti-inflammatory apolipoproteins have recently been shown to attenuate eosinophil migration and activation. Here, we focus on the anti-pathogenic properties of SCFAs and apolipoproteins on eosinophil effector function and provide insights into the potential use of SCFAs and apolipoproteins (and their mimetics) as effective agents to combat eosinophilic inflammation.

## 1. Introduction

Under steady state, eosinophil granulocytes are mainly tissue dwellers involved in the maintenance of tissue homeostasis and only make up to 5% of circulating human blood leukocytes. In healthy individuals, eosinophils have a circulating half-life of approximately 18 h and a tissue life span of at least 2–6 days. Thus, under baseline conditions, eosinophils rapidly enter several tissues, mainly the gastrointestinal tract, adipose tissue, thymus, uterus, and mammary glands, where they promote a variety of essential homeostatic functions, such as immunoregulation, tissue repair, glucose homeostasis, protection against obesity, regulation of mammary gland development, and preparation of the uterus for pregnancy (reviewed in [1]). Moreover, eosinophils promote antitumor effects in colorectal cancer [2,3,4], hepatoprotective activities [5], and cardiac protection after myocardial infarction [6].

However, specific diseases and conditions ranging from primary eosinophilic disorders, including hypereosinophilic syndrome and chronic eosinophilic leukemia [7], to infections [8], allergies [9], and chronic inflammatory diseases such as inflammatory bowel diseases [10] and eosinophilic chronic rhinosinusitis [11], can result in increased numbers of activated blood and tissue eosinophils that have the capability to cause tissue damage and dysfunction.

Under inflammatory conditions, pro-inflammatory cytokines activate eosinophils and substantially prolong their survival. As significant players in the inflammatory process, activated eosinophils are a major source of cytotoxic granule-derived proteins, such as eosinophilic cationic protein, eosinophil-derived neurotoxin, major basic protein, and eosinophil peroxidase (reviewed in [12]), and secrete an array of proinflammatory cytokines (reviewed in [13]), including interleukin (IL)-3, IL-6, and tumor necrosis factor-α. Eosinophils also produce pro-inflammatory lipid mediators, such as platelet-activating factor (PAF) and cysteinyl-leukotrienes (cysLTs) (reviewed in [14]) as well as anti-inflammatory lipid mediators including lipoxins [15], resolvins [16], and protectins [17] and release reactive oxygen species [13,18]. Eosinophils therefore promote a variety of complex immunoregulatory functions. For instance, eosinophils modulate lymphocyte recruitment and development, behave as antigen-presenting cells, are involved in T_H_2 polarization, interact with and activate other immunocompetent cells such as mast cells and macrophages, and signal to and activate resident tissue cells such as epithelial and endothelial cells (reviewed in [19]).

The management of eosinophilic inflammation strongly depends on the many individual causes of eosinophilia, but often includes corticosteroids as a first-line therapy. However, treatments with corticosteroids are not always effective and bear a broad variety of side-effects. The development of biologics, primarily targeting the eosinophil growth factor IL-5 and the IL-4/IL-13 activation axis, has been a milestone in the treatment of eosinophilic inflammation. In patients with severe eosinophilic asthma, the IL-5 antibody mepolizumab reduced annual exacerbation rates by 50% [20], and the IL-5 receptor antibody benralizumab yielded reduction rates between 17 and 51% [21]. Similarly, the anti-IL-4 receptor α antibody dupilumab reduced annual exacerbation rates by 48% in patients with uncontrolled asthma [22]. Thus, half of these patients continue to experience asthma exacerbations and suboptimal control. In the meantime, mepolizumab has been approved for the treatment of severe eosinophilic asthma, eosinophilic granulomatosis with polyangiitis, and hypereosinophilic syndrome; benralizumab has been approved for severe asthma with an eosinophilic phenotype and dupilumab has been approved for the treatment of moderate-to-severe atopic dermatitis and asthma as well as for chronic rhinosinusitis with nasal polyposis. Although biologics such as dupilumab and the novel anti-Siglec-8 antibody lirentelimab are currently tested for further indications, such as inflammatory bowel disease [23,24,25], there remain significant unmet needs for novel therapies that specifically target the cytotoxic effector functions of eosinophils without suppressing their beneficial homeostatic activities.

In recent decades, there has been a rising trend in the prevalence of allergic asthma and other allergic and chronic inflammatory diseases, especially in Western countries, which has been associated with a change in lifestyle including diet. At the same time, microbiome research has revealed that the composition of the gut microbiota significantly contributes to our well-being. For instance, anaerobic commensal bacteria produce short chain fatty acids from dietary fibers via intestinal fermentation. These SCFAs bear a broad variety of biological functions involved in the immune system, metabolism, and homeostasis and are associated with several pathophysiological conditions such as allergic inflammation, inflammatory bowel disease, and cancer [26,27,28].

Apolipoproteins (apo) are integral components of lipoproteins and play a crucial role in the synthesis and catabolism of plasma lipoproteins, in lipid transport, and as activators of certain enzymes associated with lipid and lipoprotein metabolism. Several preclinical studies have proven the anti-inflammatory properties of apolipoproteins, such as apoA-I and apoA-IV, and decreased apolipoprotein levels in patients have been linked with immune diseases such as allergies [29,30] and chronic lung inflammation (reviewed in [31,32]).

The aim of this review is to highlight the inhibitory role of SCFAs and apolipoproteins on eosinophil effector function and to discuss the therapeutic potential of SCFA and apolipoproteins for the treatment of eosinophilic diseases such as asthma, allergic rhinitis, atopic dermatitis, eosinophil esophagitis, and inflammatory bowel diseases.

## 2. Cellular Mechanism Underlying Eosinophilic Inflammation

Eosinophils arise from CD34+ multipotent hematopoietic stem cells in the bone marrow. These progenitor cells go through several intermediate stages before becoming fully mature eosinophils comprising promyeloblasts, promyelocytes, metamyelocytes, band form, and segmented form. While basal eosinophilopoiesis under homeostatic conditions is controlled by the interplay of specific transcription factors including cytosine-cytosine-adenosine-adenosine-thymidine (CCAAT)/enhancer-binding protein (C/EBP), GATA binding protein 1 (GATA-1), PU.1, and friend of GATA (FOG) (reviewed in [33]), blood and tissue eosinophilia in eosinophilic diseases is regulated by the lineage-specific cytokine IL-5 which is produced by activated type 2 T helper cells (T_H_2) cells, mast cells, or type 2 innate lymphoid cells (ILC2s). In addition, IL-3, granulocyte/macrophage colony–stimulating factor (GM-CSF), the eotaxin family (comprising the cytokine ligands (CCL) CCL11, CCL24, and CCL26), and IL-33 [34] have been shown to contribute to eosinophil differentiation. In healthy individuals, mature eosinophils exit the bone marrow, circulate in the blood for approximately 1 day, and then enter the mucosal surface of the stomach, the small, and large intestine. This tissue homing is mainly driven by the chemokine CCL11 (eotaxin-1) as well as the T_H_2 cytokines IL-5 and IL-13 and mediated by well-orchestrated steps involving eosinophil and endothelial adhesion molecules.

Thus, T_H_2 cytokines play a crucial role in regulating eosinophil tissue homing under homeostatic conditions. However, during T_H_2-driven inflammation as found in allergic diseases, the same cytokines enhance eosinophil differentiation in the bone marrow and recruit eosinophils to inflamed tissue sites leading to blood and tissue eosinophilia. Peripheral blood eosinophilia is generally defined as an absolute eosinophil count of ≥500 per microliter, whereas tissue eosinophilia and is more difficult to define. Eosinophils are terminally differentiated granulocytes, as are neutrophils and basophils, and can no longer divide. Thus, eosinophilic inflammation is the result of enhanced production in the bone marrow, tissue recruitment, and prolonged survival in response to mediators released in the environment. Besides IL-5 and CCL11, other pro-inflammatory stimuli that induce eosinophil migration are the lipid mediators prostaglandin D_2_, leukotriene (LT)B_4_, 5-oxo-eicosatetranoic acid (5-oxo-ETE), and PAF (reviewed in [35,36]), the chemokines “regulated on activation, normal T cell expressed and secreted” (RANTES, CCL5), CCL24, CCL26, monocyte chemoattractant protein-3 and -4 [37,38,39], and the complement components C3a and C5a [40]. Notably, anti-IL-5 biologics such as mepolizumab significantly reduce blood and lung eosinophilia in asthmatic patients but have no effect on the physiologic duodenal infiltration of eosinophils [41], which is, however, reduced by anti-Siglec-8 antibodies [42], suggesting that other IL-5-independent pro-survival mechanisms exist. As demonstrated in vitro, IL-3 and GM-CSF prolong eosinophil survival for weeks and prostaglandin D_2_ has been shown to maintain eosinophil survival via DP1 receptor activation [43].

During eosinophilic inflammation, eosinophils locally release their granule content via different mechanisms including piecemeal degranulation. The release of the cytotoxic cationic proteins, namely major basic protein, eosinophil peroxidase, eosinophil cationic protein, and eosinophil-derived neurotoxin, is a critical step by which eosinophils damage local structural cells leading to tissue damage and remodeling and activate other immune cells including neutrophils and mast cells. Moreover, activated eosinophils themselves are a rich source of immunomodulatory cytokines such as IL-2, -3, -4, -5, -6, -8,-10, -12, -13, -16, interferon-γ, and tumor necrosis factor-α, chemokines such as RANTES and CCL11, growth factors including GM-CSF, stem cell factor, vascular endothelial growth factor, and transforming growth factor-α (reviewed in [13]) and pro-inflammatory lipid mediators such as PAF and cysLTs (reviewed in [14]) as well as inflammation resolving lipids including lipoxins [15], resolvins [16], and protectins [17]. Eosinophil activation during inflammatory processes is also associated with increased respiratory burst and the release of reactive oxygen species, which cause endothelial dysfunction and tissue damage and therefore play an important role in the progression of inflammatory diseases [18]. Moreover, eosinophil extracellular traps have been noted in several eosinophil-associated disorders including eosinophilic asthma [44] and chronic rhinosinusitis [45]. Eosinophil extracellular traps, which are composed of mitochondrial DNA and granule proteins, contribute to the viscosity of secretions and are capable of activating both the innate and adaptive immune systems [46,47].

Thus, eosinophils are pluripotent effector cells involved in the pathophysiology of various inflammatory diseases. In most eosinophilic diseases, eosinophil counts are associated with disease progression and a poor prognosis. For instance, patients with moderate to severe allergic rhinitis are characterized by elevated levels of activated and pathogenic eosinophils, which are associated with higher production of eosinophil cationic protein, eosinophil peroxidase, and IL-4 in the peripheral blood [48]. Similarly, eosinophilic asthma has been identified as a distinct phenotype of severe asthma often associated with frequent exacerbations and a weak treatment response (reviewed in [49]). The increased presence of eosinophils in peripheral blood and inflammatory infiltrates of atopic dermatitis patients has long been established and eosinophil numbers as well as eosinophil granule protein levels in peripheral blood appear to correlate with disease activity [50]. Eosinophilic chronic rhinosinusitis is a subgroup of chronic rhinosinusitis with nasal polyps, which is associated with severe eosinophilic infiltration and persistent symptoms such as dyssomnia, nasal obstruction, and discharge [11]. Eosinophilic esophagitis, which is caused by an adaptive immune response primarily to food antigens, is characterized by a chronic eosinophilic inflammation leading to an altered esophageal barrier function and is associated with chronic food dysphagia and food impaction (reviewed in [51]). In inflammatory bowel disease, comprising ulcerative colitis and Crohn’s disease, eosinophils have been considered as pro-inflammatory cells associated with increased tissue damage. However, a recent study suggested that inflammatory bowel disease patients with eosinophil-predominant inflammation might have a reduced risk of disease flares and hospitalization [52].

## 3. Apolipoproteins

Apolipoproteins are amphipathic lipid-binding molecules involved in the transport of cholesterol, triglycerides, and phospholipids. They are an integral part of lipoprotein particles and are important in stabilizing their structure. Evidence from numerous studies has shown that apolipoproteins play a vital role in cardiovascular disease, such as atherosclerosis and coronary artery disorders [53,54,55]. Moreover, a number of recent reports have linked apolipoproteins, especially those of the apoA-I/C-III/A-IV/A-V gene cluster, with various types of immune diseases such as cancer (reviewed in [56]), allergy [29,30], and chronic lung inflammation (reviewed in [31,32]).

### 3.1. Apolipoprotein A-I

Apolipoprotein A-I, which is synthesized by hepatocytes and enterocytes and secreted into the blood, represents the principal structural and functional protein constituent of high-density lipoprotein (HDL). HDL exerts various anti-atherogenic features, including reverse cholesterol transfer, anti-oxidative, anti-thrombotic, vasodilatory, and endothelial protective properties [57,58,59]. Notably, these anti-atherogenic functions of HDL have largely been attributed to apoA-I. ApoA-I interacts with its cellular receptor, the ATP-binding cassette subfamily A, member 1 (ABCA1), to facilitate cholesterol efflux from cells to form nascent HDL particles. The ability of apoA-I to promote cholesterol efflux from cells that mediate adaptive immunity, such as macrophages [60], can attenuate their activation. Thus, besides its crucial role in reverse cholesterol transport, apoA-I was shown to promote regulatory and inhibitory effects on inflammatory processes through several pathways [61,62,63].

### 3.2. Apolipoprotein A-IV

Apolipoprotein A-IV is another component of the A-I/C-III/A-IV/A-V gene cluster which is synthesized in the intestine and liver and secreted into the mesenteric lymph on chylomicrons. Most apoA-IV molecules dissociate into plasma and only 25% are taken up by HDL [64]. Many physiological functions have been attributed to apoA-IV, suggesting that the biological role of apoA-IV is much broader than just lipid metabolism [65,66] and reverse-cholesterol transport [67]. For instance, apoA-IV was identified as an acute satiety factor, regulator of gastric function, and modulator of glucose homeostasis (reviewed in [68]). Further, atheroprotective [69,70], anti-oxidant [71], and anti-inflammatory properties have been associated with apoA-IV [29,72].

The following section highlights the evidence for the anti-inflammatory and beneficial role of apoA-I and apoA-IV in eosinophil cellular function and eosinophilic diseases.

### 3.3. Effects of Apolipoproteins on Eosinophil Cellular Function

**Figure 1:**
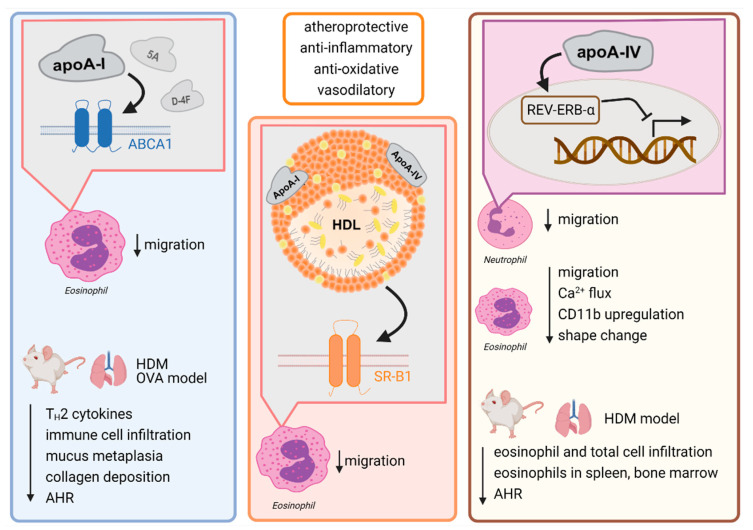
HDL and its components apolipoprotein A-I and apolipoprotein A-IV exhibit immunomodulatory actions via its receptors leading to amelioration of allergic inflammation. Abbreviations: ABCA1, ATP binding cassette subfamily A member 1; AHR, airway hyperresponsiveness; apoA-I, apolipoprotein A-I; apoA-IV, apolipoprotein A-IV; Ca^2+^, calcium; HDL, high-density lipoprotein; HDM, house dust mite; OVA, ovalbumin; SR-B1, scavenger receptor class B type 1.

In the past, several studies investigated the anti-inflammatory effects of apoA-I and apoA-I mimetic peptides on the in vitro function of leukocytes such as neutrophils [73,74,75,76,77,78], monocytes [79,80,81,82,83,84], and macrophages [85,86,87,88,89,90]; however, thus far, little is known about their impact on eosinophil effector function. Just recently, a study from Roula et al., revealed that eosinophil migration in response to CCL11 is impaired by pretreatment with apoA-I in an ABCA1 dependent manner, whereas HDL-induced inhibition is mediated by the HDL receptor scavenger receptor class B, type 1 (SR-B1) [29]. Moreover, it was demonstrated that apoA-IV significantly inhibits human neutrophil chemotaxis in response to IL-8 [29]. Further, apoA-IV potently decreased eosinophil responsiveness as measured by calcium (Ca^2+^)-flux, shape change, integrin (CD11b) expression, and chemotaxis. The newly discovered underlying molecular mechanism was independent of ABCA1 and SR-B1 and involved the activation of the nuclear receptor and transcriptional repressor Rev-ErbA-α (NR1D1) and induced a PI3K/PDK1/PKA-dependent signaling cascade [29]. An overview of the immunomodulatory actions of apoA-I and -IV and the proposed molecular mechanisms is given in Figure 1.

### 3.4. The Role of Apolipoproteins in Mouse Models of Eosinophilia-Associated Diseases

#### 3.4.1. Mouse Models of Allergic Lung Inflammation

Experimental results from murine models of allergic airway inflammation revealed a protective role for apoA-I in the pathogenesis of asthmatic diseases. First, reduced apoA-I levels were determined in lung parenchyma extracts from ovalbumin (OVA)-challenged mice [91]. Appropriately, the OVA challenge of apoA-I knockout mice led to increased neutrophilic lung inflammation associated with increased type-I cytokines in a granulocyte-colony stimulating factor (G-CSF)-dependent manner [92]. Moreover, lipopolysaccharide (LPS)-challenged apoA-I knockout mice showed increased numbers of bronchoalveolar lavage (BAL) fluid neutrophils compared with wild-type mice, whereas the apoA-I mimetic peptide L-4F reduced airway neutrophilia [79]. Besides neutrophil inflammation, apoA-I also showed positive effects on airway eosinophilia. Systemic administration of the apoA-I mimetic peptide 5A significantly decreased the number of BAL fluid eosinophils, lymphocytes, and neutrophils in a house dust mite model [93]. The prevented airway inflammation was associated with decreased T_H_2 cytokines, macrophage activation, airway hyperreactivity, and mucus metaplasia [93]. Further, intranasal administration of the apoA-I mimetic peptide D-4F led to decreased peripheral blood eosinophils, EPO activity in BAL fluid, airway hyperreactivity, lung collagen deposition, as well as total immunoglobulin (Ig) E, and p-HDL in plasma in an OVA mouse model [94]. Moreover, intranasal administration of human apoA-I to house dust mite-challenged mice reduced airway inflammation, with decreases in BAL fluid eosinophils, neutrophils, lymphocytes, and macrophages, as well as airway hyperreactivity and lung levels of cytokines such as IL-33, and increased airway epithelial cell tight junction proteins, as well as levels of lipoxin A_4_ [95].

Similar to apoA-I and apoA-I mimetic peptides, intraperitoneal application of human apoA-IV ameliorated eosinophilic airway inflammation as observed by reduced eosinophil counts in the BAL fluid in house dust mite-challenged mice and prevented airway hyperreactivity as assessed by reduced airway resistance and improved airway compliance [29].

#### 3.4.2. Mouse Models of Inflammatory Bowel Disease

The intestine and liver are the major organs responsible for apolipoprotein synthesis. Dysfunction due to chronic inflammation is associated with a decrease in levels of HDL cholesterol and apoA-I [96,97]. Moreover, apoA-I mimetic peptides selectively target the small intestine, where they are reabsorbed by the intestinal mucosa, and mediate the transintestinal efflux of cholesterol [98]. Thus, during recent years, multiple experimental studies investigated the anti-inflammatory role of apoA-I and apoA-I mimetics in models of inflammatory bowel disease.

Gerstner et al., showed that dextran sulfate sodium (DSS)/2,4,6-trinitrobenzene sulfonic acid (TNBS)-treated apoA-I knockout mice displayed increased mucosal damage, increased intestinal myeloperoxidase (MPO) activity, and mRNA expression of tumor necrosis factor-α and intercellular adhesion molecule-1 as compared with wild type and apoA-I transgenic mice. In contrast, apoA-I transgenic mice had less severe symptoms and MPO activity in both the DSS and TNBS colitis models [99]. Gkouskou and colleagues reported that apoA-I is expressed at higher levels in the proximal part of the colon and its ablation resulted in exaggerated DSS-induced colitis and azoxymethane /DSS-induced colon tumors. Treatment with the apoA-I mimetic peptide D-4F ameliorated the manifestations of both diseases [100]. Similarly, D-4F also mitigated intestinal inflammation in cyclooxygenase (COX) 2-/myeloid-knockout mice fed with a cholate-containing high-fat diet and in a piroxicam-accelerated IL-10 knockout model of inflammatory bowel disease [101]. Further, Nowacki et al. showed that treatment with the 5A mimetic peptide potently reduced DSS colitis as indicated by improved disease activity, colon histology as well as decreased intestinal MPO and plasma cytokine levels. Moreover, the number of activated intestinal monocytes was decreased in 5A peptide-treated mice [102].

In addition, Vowinkel et al., examined the anti-inflammatory effect of systemic application of apoA-IV in a mouse model of DSS-induced acute colitis [72]. ApoA-IV significantly delayed the onset and reduced the severity of DSS-induced inflammation as assessed by clinical disease activity score, histology, and MPO activity. Moreover, apoA-IV significantly inhibited leukocyte and platelet adhesive interactions. Accordingly, apoA-IV knockout mice exhibited a significantly greater inflammatory response to DSS than did their wild type littermates. The administration of apoA-IV reversed this greater susceptibility to DSS-induced inflammation.

### 3.5. The Role of Apolipoprotein A-I in Eosinophilia-Associated Diseases

Just recently, a cross-sectional, longitudinal analysis of the UK Biobank investigated the relationship of blood lipid and lipoprotein levels with leukocyte counts and revealed an association of higher eosinophil counts with lower HDL cholesterol and apoA-I concentrations [103]. Evidence for the association of apolipoproteins and eosinophilic diseases such as allergic rhinitis, asthma, atopic dermatitis, and inflammatory bowel disease is summarized below.

#### 3.5.1. Allergic Rhinitis

Interestingly, several studies indicate a local increase in apolipoproteins, especially apoA-I, in the nasal fluid of rhinitis patients. It is likely that apolipoproteins accumulate in the paranasal sinuses due to increased vascular permeability; however, it cannot be excluded that they are also released locally by infiltrating inflammatory cells.

A study from 2010 investigated changes in the nasal lavage fluid proteome after allergen challenge of potassium persulfate sensitized allergic rhinitis patients. As a major finding, increased abundance of apoA-I was detected post-challenge solely in the group of symptomatic patients [104]. In further proteome studies, apoA-I was significantly upregulated in the nasal mucus of allergic rhinitis patients, indicating a direct modulation of the local immune response by apoA-I [105,106]. In a study from Trakaki and colleagues, a markedly altered HDL composition was found in serum from untreated allergic rhinitis patients. Beside other alterations, apoA-I was significantly decreased in HDL of allergic patients compared to healthy controls [30].

Makino et al. revealed that serum levels of apoA-IV significantly increased in allergic rhinitis patients treated with sublingual immunotherapy but not in placebo-treated patients [107]. Furthermore, apoA-IV correlated with the clinical symptom-medication scores and with quality-of-life scores of sublingual immunotherapy-treated patients. In addition, data from Roula et al. indicated a significant decrease in apoA-IV serum levels in patients with symptoms of allergic rhinitis compared to healthy controls [29].

In a previous report, Tomazic et al., revealed the presence of apoA-IV in mucus samples of allergic rhinitis patients [43,108]. Similar to allergy, chronic rhinosinusitis is characterized by a pronounced eosinophilic inflammation of the lining of the nose and paranasal sinuses. Thus, Roula et al. assessed apoA-IV mucus levels in patients with chronic rhinosinusitis and found apoA-IV mucus levels to be significantly increased in patients suffering from chronic rhinosinusitis with nasal polyps [29]. Interestingly, apoA-IV levels also correlated with the histology score and radiologic Lund-Mackay score [29].

#### 3.5.2. Asthma

Although contradictory data exist, most studies indicate that decreased apoA-I levels are associated with an increased risk of asthma, whereas higher apoA-I levels are associated with less severe airflow obstruction in asthmatics. In a study from 2006, Ekmekci and colleagues analyzed the lipoprotein and apolipoprotein plasma profile from bronchial asthma patients but could not detect any differences in HDL and apoA-I levels between asthmatics and healthy controls [109]. However, a proteome analysis from nasal lavage fluid from asthmatic patients suggested an association between eosinophil cationic protein and apoA-I levels during the early response in aspirin-exacerbated respiratory disease patients [110]. Interestingly, a proteome study in BAL fluid from asthma patients revealed that apoA-I was detected in all patient samples but none of the healthy controls [111]. Similarly, a population-based cross-sectional study among school children suggested an association of high plasma apoA-I concentrations with a higher prevalence of wheeze, and a trend with asthma was observed [112]. This is in contrast to a study from Cirillo et al., who reported a positive correlation of serum apoA-I levels with less severe airflow obstruction in asthmatic individuals [113]. This observation was confirmed by Barochia and colleagues who indicated that serum levels of HDL cholesterol and apoA-I positively correlate with the forced expiratory volume in one second in subjects with allergic asthma [114].

#### 3.5.3. Atopic Dermatitis

Studies assessing the impact of apolipoproteins on the outcome of atopic dermatitis are still missing. However, high expression levels of apoA-I were demonstrated in the horny layer of skin from atopic dermatitis patients in comparison to healthy controls. Moreover, apoA-I expression correlated with the severity of specific eruptions [115].

#### 3.5.4. Inflammatory Bowel Disease

Increasing evidence further suggests that changes in the apolipoprotein profile might be involved in the pathophysiology of inflammatory bowel diseases. An investigation in inflammatory bowel disease patients revealed significantly lower serum cholesterol, HDL-cholesterol, apoA-I, apoC-II, apoC-III bound to apoB, phospholipids, and phospholipids not bound to apoB levels, and apoA-I immunoreactivity compared to healthy controls [97]. Further, a characterization of the global pattern of ileal gene expression and ileal microbial community in treatment-naive pediatric patients with Crohn’s disease, patients with ulcerative colitis, and control individuals revealed that apoA-I gene expression was downregulated and associated with Crohn’s disease-specific alterations in Firmicutes abundance. “The decreased apoA-I gene expression favored oxidative stress and Th1 polarization and was maximally altered in patients with more severe mucosal injury” [116]. Moreover, a regression model that included apoA-I gene expression and microbial abundance more accurately predicted steroid-free remission than a model using clinical factors alone [116]. Moreover, apoA-I, apoA-IV, apoA-B, and apoC-III seem to be associated with different lesion sites, including the colon and ileum, and are highly expressed in the colon of ulcerative colitis and Crohn’s disease patients [117].

In patients with Crohn’s disease, univariate and multiple logistic regression analysis revealed an association between apoA-IV plasma levels, C-reactive protein, and disease activity. In ulcerative colitis the apoA-IV gene variant 360 His but not apoA-IV levels were associated with increased disease activity in univariate analysis, whereas this association was lost in multiple logistic regression analysis [118].

## 4. Short Chain Fatty Acids

Short chain fatty acids such as formate, acetate, propionate, butyrate, isobutyrate, valerate, isovalerate, and 2-methylbutanoate are saturated, aliphatic fatty acids are produced by anaerobic commensal bacteria via intestinal saccharolytic fermentation of saccharides and indigestible foods such as dietary fibers. Thus, the production of SCFAs strongly depends on the diversity of the gut microbiota and diet composition. Accordingly, only low concentrations of SCFAs are found in the small intestinal and caecal content of germ-free mice and rats [118]. In humans, the most abundant SCFAs in the colon are acetate, propionate and butyrate [119]. Of note, Faecalibacterium prausnitzii is the major butyrate-producing bacterium of the human intestine with suggested anti-inflammatory properties [120,121]. Moreover, several probiotic microorganisms have been shown to promote anti-inflammatory effects by enhancing the production of SCFAs, particularly of butyrate (reviewed in [122]).

SCFAs are absorbed by the intestinal lumen [123] or taken up by enterocytes via sodium-coupled monocarboxylate transporter-1 and monocarboxylate transporter-1 to use them as an energy source [124]. Notably, SCFA transporters are also found in immune cells such as lymphocytes, monocytes, neutrophils, and eosinophils [125]. SCFAs that have not been absorbed or utilized by enterocytes are excreted or enter the circulation via the portal vein and are distributed to peripheral tissues or reach the liver where they are metabolized by hepatocytes [126,127]. Compared to the large intestine, only low SCFA concentrations can be found in the circulation, with acetate being the most abundant member [128].

SCFAs bear a broad variety of biological functions involved in the immune and nervous system, in metabolism, and gut homeostasis [129]. Moreover, SCFAs are associated with several pathophysiological conditions such as obesity and allergic inflammation, inflammatory bowel disease, and cancer [26,28,130,131]. SCFAs mediate their effects by inhibiting histone deacetylases (HDAC) or by engaging with the G protein coupled receptors (GPR)43, also known as free fatty acid receptor (FFA)2 and GPR41, also known as FFA3 [132,133,134]. GPR43 is a G_q/11_ or G_i_-coupled receptor [132] which is found in several tissues and cell types but most abundantly expressed in immune cells such as neutrophils, monocytes, lymphocytes, and eosinophils [132,133,135]. GPR41 is a G_i_-coupled receptor [132] with the highest expression in the epithelium of the colon but also found in the spleen, pancreas, and the lungs [136,137]. Recently, GPR41 expression has been confirmed on the mRNA level in peripheral blood eosinophils [138] and in a subset of T_H_2 cells in eosinophilic esophagitis (EoE) [139].

Short chain fatty acids as HDAC inhibitors. The SCFAs butyrate, acetate, and propionate act as an HDAC inhibitor with butyrate being the most potent candidate [140,141]. In general, HDACs are a class of enzymes that remove acetyl groups from chromatin, thereby repressing transcription [142] and regulating a broad spectrum of cellular functions such as migration, metastasis [143,144], and survival [145,146]. Of note, butyrate is being discussed as an anti-neoplastic treatment in colorectal cancer due to its ability to inhibit cancer cell proliferation, angiogenesis, and metastasis [147]. Moreover, the HDAC inhibitor trichostatin A was shown to induce apoptosis in human eosinophils [145] and seems to have beneficial effects in mouse models of allergic lung inflammation [148,149].

### 4.1. Effects of SCFA on Eosinophil Cellular Function

In many protocols, butyrate is used to differentiate HL-60 and EoL-1 leukemic cell lines into an eosinophilic phenotype [150,151] and to induce IL-5 receptor expression [152]. As butyrate inhibits histone deacetylases and other histone deacetylase inhibitors such as apicidin are also able to differentiate EoL-1 cells, continuous acetylation of histones H4 and H3 followed by inhibition of EoL-1 proliferation and the induction of markers of mature eosinophils such as integrins and CCR3 receptors seems to be the responsible mechanism for this effect [153].

In a recent publication, Theiler et al. showed for the first time that both acetate and propionate bind to GPR43 in human eosinophils leading to an increase in intracellular Ca^2+^ increase, whereas butyrate had no effect on Ca^2+^ flux under the same conditions. Furthermore, both acetate and propionate stimulated the production of reactive oxygen species, whereas butyrate again was ineffective [154]. Conversely, propionate and butyrate induced the intrinsic apoptosis pathway in human eosinophils in a GPR43- and GPR41-independent manner, most likely mediated through HDAC inhibition, whereas acetate did not impair eosinophil survival [154].

In further experiments, integrin α-4 (CD49d) transcription was significantly reduced by all three SCFAs, whereas at the protein level CD49d surface expression was only decreased by propionate and butyrate. Expression of the extracellular matrix receptor CD44 mRNA was decreased by butyrate but was unaffected by propionate. Expression of CCR3 was downregulated by propionate but the effect of butyrate was more pronounced. In contrast, acetate did not blunt CCR3 or CD44 mRNA. Additionally, butyrate reduced CCR3 and CD44 protein levels on eosinophils; however, acetate and propionate were not able to mimic this effect. L-selectin surface expression on eosinophils was unaffected by all SCFAs [154].

**Figure 2:**
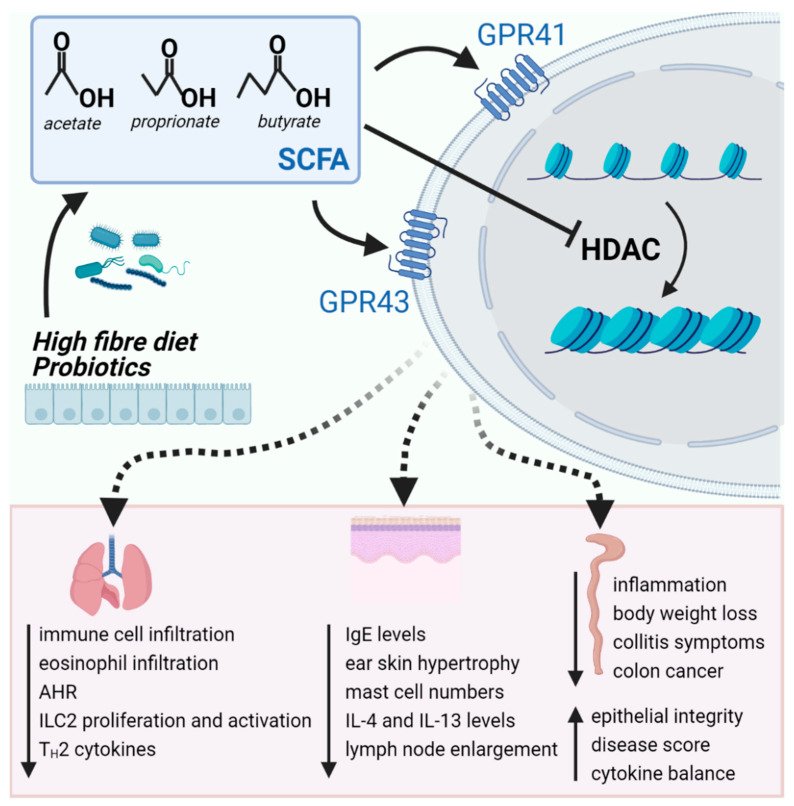
Short chain fatty acids produced by the gut microbiota bind to its receptors and/or act as histone deacetylase inhibitors reducing inflammation in the lungs, skin and gut. Abbreviations: AHR, airway hyperresponsiveness; HDAC, histone deacetylase; IgE, immunoglobulin E; ILC2, type 2 innate lymphoid cells; SCFA, short chain fatty acids.

Theiler et al., also investigated whether SCFAs impair the migratory responsiveness of eosinophils and found that propionate and butyrate prevent eosinophil adhesion to endothelial monolayers in response to eotaxin-2 (CCL24). In a chemotaxis assay, butyrate, but not propionate and acetate, attenuated eosinophil migration toward CCL24. Pretreatment of eosinophils with the pan-HDACi TSA mimicked the effect of butyrate and similarly inhibited adhesion and migration [154]. The immunomodulatory actions of SCFAs and the involved cellular mechanisms are summarized in Figure 2.

### 4.2. The Role of SCFAs in Mouse Models of Eosinophilia-Associated Diseases

#### 4.2.1. Mouse models of Airway Inflammation

To ascertain the relevance of a diverse microbiome on the development of allergic diseases, Herbst et al. used a model of OVA-induced allergic airway inflammation in germ-free mice, resulting in increased numbers of airway-infiltrating lymphocytes, eosinophils and dendritic cells, elevated IgE, and increased local production of T_H_2-associated cytokines compared with control specific-pathogen-free mice. Interestingly, this increase could be reversed by recolonization of germ-free mice with the complex commensal flora of specific-pathogen-free mice [155].

In addition, several mouse models of allergic airway inflammation have highlighted the positive effects of high-fiber diet, pre-, and probiotics on the inflammatory response and airway hyperresponsiveness by changing the gut microbiota and elevating the levels of SCFAs [130,156,157,158,159]. Further, Trompette et al., showed that application of propionate, alone, mimicked the positive effect of the high-fiber diet and protected against house dust mite-induced allergic lung inflammation in a GPR41-dependent manner [160]. A similar study from Thorburn et al., validated that maternal high-fiber or acetate intake prevents the development of house dust mite-induced allergic lung inflammation in adult offspring [130]. Cait et al., confirmed that dysbiotic mice have a higher susceptibility to OVA-induced airway inflammation which can be prevented by oral application of SCFAs (mixture of acetate, propionate and butyrate) [161]. SCFAs-treated mice showed decreased levels of circulating IgE, reduced leukocyte infiltration into the airways and especially improved airway eosinophilia in response to OVA [161]. Of note, the authors showed that butyrate supplementation, alone, was sufficient to attenuate OVA-induced airway inflammation [161]. Recently, Thio et al. focused on the immunomodulatory role of butyrate in the regulation of ILC2-mediated airway hyperresponsiveness and inflammatory parameters in a model of Alternaria alternata-induced allergic inflammation [162]. As a result, oral or intranasal application of butyrate improved airway eosinophilia and lung function by inhibiting the proliferation and cytokine production of ILC2s in a receptor-independent manner via HDAC inhibition [162]. Similarly, in order to address whether butyrate can modulate lung inflammation in the absence of T cells, recombination activating gene 2 protein knockout (RAG2^−/−^) mice were treated with butyrate following an intranasal application of IL-33 to mimic allergic lung inflammation [163]. Interestingly, direct butyrate treatment significantly affected ILC2 function in vitro and in vivo. The authors also confirmed that Clostridia butyricum provides systemic butyrate to dampen ILC2-dependent airway hyperresponsiveness [163].

In a recent study, Theiler and colleagues investigated the direct effects of systemic butyrate treatment on acute OVA-induced lung inflammation. Butyrate significantly reduced airway eosinophilia and T_H_2 cytokines present in the BAL fluid, and completely reversed the OVA-induced airway hyperresponsiveness. Moreover, an increase in IL-5 receptor expression and a trend towards an apoptotic phenotype in lung eosinophils was detected [154].

#### 4.2.2. Mouse Models of Atopic Dermatitis

During the last decade, several studies pointed out the beneficial and anti-inflammatory effects of a high-fiber diet and prebiotics such as poly- and oligosaccharides in murine models of atopic dermatitis [164,165,166,167]. Besides dietary fibers, the impact of probiotic supplementations on experimental atopic dermatitis was intensively studied, revealing a changed gut microbiota and the associated increase in SCFAs as a possible therapeutic mechanism [168,169,170,171].

Of note, Kang et al. presented novel data suggesting a beneficial role for the FFA2 (GPR43) agonist, 4-chloro-α-(1-methylethyl)-N-2-thiazoylylbenzeneacetanilide (4-CMTB), in a mouse model of 2,4-dinitrochlorobenzene (DNCB)-induced atopic dermatitis. Treatment with 4-CMTB significantly suppressed IgE levels, ear skin hypertrophy, mast cell accumulation, IL-4 and IL-13 levels in the ears, and DNCB-induced lymph node enlargement [172].

#### 4.2.3. Mouse Models of Inflammatory Bowel Disease

During the last twenty years, multiple publications identified a changed composition and metabolism of the gut microbiota associated with increased SCFA concentrations as a possible anti-inflammatory mechanism [173,174,175,176,177,178,179,180]. In contrast, high-sugar diet was shown to enhance susceptibility to DSS-colitis via depletion of luminal short chain fatty acids [181]. Similar to dietary fibers, several probiotics and symbiotic supplementations with dietary fibers also ameliorated disease activity, changed the gut microbiota, and showed SCFA-supporting effects [182,183,184,185,186,187,188,189,190]. These promising reports inspired further studies on the direct effect of SCFA supplementation on experimental colitis.

In 2013, Mishiro and colleagues demonstrated that the intrarectal administration of butyrate leads to attenuated intestinal inflammatory parameters and inhibited body weight loss in the murine DSS-model. The authors further suggested, that acetylation on histone 3 lysine 9 (acetyl-H3K9) around the MFG-E8 promoter is involved in the butyrate-mediated anti-inflammatory effect [191]. In a study from 2017, Simeoli et al., investigated the therapeutic impact of the butyrate-releasing derivative N-(1-carbamoyl-2-phenylethyl) butyramide (FBA) in an experimental DSS-model. FBA treatment reduced colitis symptoms and colon damage, decreased inflammatory cell infiltration, and reversed the imbalance between pro- and anti-inflammatory cytokines. Moreover, FBA restored the deficiency of the butyrate transporter and improved intestinal epithelial integrity. FBA, similar to its parental compound sodium butyrate, inhibited histone deacetylase-9, inhibited nuclear factor kappa B (NF-κB), and up-regulated peroxisome proliferator activated factor-γ [192]. Likewise, Lee and colleagues studied the effects of oral butyrate administration and observed a similar improvement in the disease score. In immunohistochemical staining, IκBα phosphorylation was attenuated, and histone H3 acetylation was reversed in the treated colons, indicating that oral supplementation with butyrate attenuates experimental murine colitis by blocking NF-κB signaling and reverses histone acetylation [193]. In 2018, Chen et al., assessed the anti-inflammatory activity of butyrate in a TNBS-induced mouse model that resembles Crohn’s disease, and showed that butyrate significantly ameliorated the inflammatory response and intestinal epithelium barrier dysfunction. Their data further indicated that butyrate ameliorated the TNBS-induced inflammatory response through activating GPR109A and inhibiting the AKT and NF-κB p65 signaling pathways [194]. In the same year, Tian et al. investigated the direct effect of oral administration of a mixture of short chain fatty acids on azoxymethane/DSS-induced colitis-associated colorectal cancer. SCFAs significantly reduced the tumor incidence and size by improving colon inflammation and disease activity index score, as well as suppressing the expression of proinflammatory cytokines [195].

#### 4.2.4. Mouse Models of Eosinophilic Esophagitis

Probiotics, prebiotics, and SCFAs have been extensively studied and tested in various allergy models. However, most reports are limited to allergic airway inflammation or atopic dermatitis. Thus, Holvoet and colleagues are the first who described the beneficial effect of the probiotic Lactococcus lactis NCC 2287 in a murine model of eosinophilic esophagitis. Oral therapeutic treatment with L. lactis NCC 2287 significantly decreased esophageal and bronchoalveolar eosinophilia induced by epicutaneous sensitization with Aspergillus fumigatus protein extract [196].

### 4.3. The Role of SCFAs in Eosinophilia-Associated Diseases

Dietary fibers, when fermented by the gut microbiota, are the major source of SCFAs. Although dietary fibers are present in a broad range of food sources, a reduction in fiber intake is inherent in changes from traditional to Western food models [197]. The association between fiber intake and health first came to public attention in the 1970s, due to the high prevalence of diseases such as diverticulitis, constipation, or hemorrhoids in economically developed countries, not existing in less industrialized countries [198]. In 2010, De Filippo et al., found that the fecal microbiota of children from rural Africa, who consume a diet with a natural high fiber content, showed a significant enrichment of bacteria associated with fiber fermentation and contained significantly more SCFAs compared to children living in the European Union [199].

The section below gives an overview of the possible beneficial effects of pro-, prebiotics, and SCFAs on the most common eosinophilic diseases.

#### 4.3.1. Asthma

Arrieta et al., compared the gut microbiota of infants enrolled in the Canadian Healthy Infant Longitudinal Development (CHILD) study, and showed that children at risk of asthma exhibited transient gut microbial dysbiosis during the first months of life [200]. Interestingly, another study pointed out that the early microbiome of children who develop allergic sensitization later in childhood lacked genes encoding enzymes for carbohydrate degradation and butyrate formation [201]. Just recently, a significant decrease in total fatty acids and specifically in acetate, propionate and butyrate has been established in the feces of patients with bronchial asthma regardless of the phenotype [202]. Accordingly, children with high levels of butyrate or propionate in feces at the age of one year have significantly less atopic sensitization and are less likely to have allergic asthma, food allergy, or allergic rhinitis between 3 and 6 years [203]. A study from Berthon et al., revealed that adult patients with severe persistent asthma consumed more fat and less fiber compared to healthy controls. Among asthmatics, high fat and low fiber intake was associated with reduced lung function and airway eosinophilia [204].

The beneficial impact of fiber intake on asthma outcome was further supported by a study investigating the acute effect of a single intake of soluble fiber on asthma parameters. Four hours after meal consumption, total sputum total cell count, neutrophils, macrophages, lymphocytes, sputum IL-8, and exhaled nitric oxide significantly decreased. Moreover, GPR41 and GPR43 sputum gene expression increased and lung function significantly improved [205]. In a recent study by McLoughlin et al., stable asthmatic patients received three times daily for seven days an oral intervention of soluble fibers, soluble fibers plus probiotics, or placebo. Following the soluble fiber intervention there was an improvement in the asthma control questionnaire, sputum eosinophils decreased, and sputum histone deacetylase 9 gene expression decreased [206].

#### 4.3.2. Atopic Dermatitis

Multiple studies showed an association between the diversity of the skin microbiome and the development atopic dermatitis [207,208], whereas little is known about the role of the gut microbiota in the pathogenesis of atopic dermatitis and its possible beneficial impact still remains controversial [209,210]. Similarly, despite positive reports, the usefulness of pre- and probiotics on the prevention and treatment of eczema and atopic dermatitis is still a matter of debate [211,212,213,214,215,216].

Nylund and colleagues found that the severity of eczema correlated inversely with intestinal microbiota diversity and abundance of butyrate-producing bacteria in infants with atopic dermatitis, suggesting that butyrate-producing bacteria play a role in alleviating symptoms of atopic eczema [217]. Similarly, Song et al., reported that enrichment of a subspecies of the major gut species Faecalibacterium prausnitzii is strongly associated with atopic dermatitis. Moreover, by investigating fecal samples, the authors found decreased levels of butyrate and propionate in their cohort of atopic dermatitis patients [218]. In a study of Matsumoto and colleagues, a probiotic yogurt containing Bifidobacterium animalis subsp. lactis LKM512 improved symptoms such as itch and burning and led to an enrichment of the gut microbiota with bacterial species and phylotypes of Bifidobacterium, Clostridium cluster IV and subcluster XIVa. Moreover, fecal spermidine and butyrate also increased [219]. Recently, Traisaeng et al. demonstrated that a derivate of butyric acid inhibits the growth of Staphylococcus aureus isolated from patients with atopic dermatitis [220].

In contrast, in a study from 2012, Roessler et al., found no differences in fecal concentrations of total short chain fatty acids as well as the levels of acetate, propionate, butyrate, valerate, and caproate after probiotic supplementation in both healthy subjects and atopic dermatitis patients [221].

#### 4.3.3. Inflammatory Bowel Diseases

A first protective effect of a high-fiber diet on inflammatory bowel disease was pointed out in 1973 by Burkitt, who worked in African countries and observed a low incidence of colon cancer and other inflammatory intestinal diseases among the inhabitants, whose diet was normally rich in dietary fibers [222]. Since then, several studies discussed the positive effects of high-fiber diet containing oligo- and polysaccharides on disease activity and quality of life, especially in patients with ulcerative colitis [223,224,225,226]. Just recently, a cross-over study of patients with ulcerative colitis in remission by Fritsch et al. showed that low-fat and high-fiber diet improves the overall quality of life of patients [227]. However, during disease exacerbation, a low fiber diet is recommended for most patients and it is commonly observed that patients with inflammatory bowel disease are permanently avoiding dietary fibers, regardless of disease activity [228,229]. Similar to dietary fibers and prebiotics, the therapeutic potential of probiotics which should restore the enteric microbiota and support SCFA synthesis, was investigated in multiple trials [185,230,231,232,233]. Two meta-analysis of studies in ulcerative colitis patients demonstrated therapeutic benefits of probiotics over placebo [230,231]. However, most available studies are biased by several drawbacks, including small samples and poor methodological quality [234].

Since the late 1980s/early 1990s, multiple studies pointed out the association between inflammatory bowel diseases, dysbiosis of the gut microbiota, and a decreased production of SCFAs [131,235,236,237,238,239,240,241]. Moreover, several studies indicated a beneficial effect of topic SCFA treatment for patients with ulcerative colitis. In 1991, Breuer et al., published a preliminary report of ulcerative colitis patients using rectal irrigations with an acetate, propionate, and butyrate containing solution. Of the 10 patients who completed the trial, nine were much improved and showed a change in disease activity score and mucosal histology score, indicating that ulcerative colitis patients benefit from increased contact with these critical energy substrates [242]. Although a follow-up study only showed beneficial effects of SCFA treatment in a sub-group of patients [243], the promising results of topical SCFA were confirmed in several other studies [244,245]. In 1996, Steinhart and colleagues examined the therapeutic potential of butyrate in patients with distal ulcerative colitis, however, the results suggested that butyrate is not efficacious for the treatment of distal ulcerative colitis [246]. In a study from Assisi et al., 216 patients with ulcerative colitis who showed an incomplete response to standard mesalazine treatment were treated with a combined formulation of mesalazine, butyrate, and inulin. The results obtained indicated that the addition of butyrate and inulin was effective in reducing disease activity, with a marked improvement of symptoms, and in the endoscopic appearance of the mucosa [247]. In a recent study by Magnusson and colleagues, biopsies from ulcerative colitis patients were cultivated with or without butyrate, cytokines were measured in supernatants and mRNA gene expression was analyzed. Of note, the authors showed that butyrate more potently down-regulates the gene expression of inflammatory pathways in non-inflamed controls than in inflamed tissue of ulcerative colitis patients. These discrepancies may at least partly explain why the anticipated anti-inflammatory effects of local butyrate induction or supplementation are not always obtained [248].

#### 4.3.4. Eosinophilic Esophagitis

Similar to other eosinophilia-associated diseases, microbiome research in the field of eosinophilic esophagitis has increased in recent years. On the one hand, several reports indicated that patients with EoE have a certain esophageal microbiome distinct from that of non-EoE controls [249,250,251,252,253,254,255]; however, thus far the causal relationship that regulates these changes is still uncertain (reviewed in [256]). On the other hand, no significant differences in the esophageal microbiome were observed between newly diagnosed EoE cases and non-EoE controls in adults, while use of proton pump inhibitors was significantly associated with five taxa including Absconditabacteria at the phylum level and Burkholderia at the genus level [257]. Thus, future studies in newly diagnosed and treated children and adults are needed to unravel the association between the esophageal microbiome and the development of EoE. Of note, dietary fiber intake seems to be associated with a distinct esophageal microbiome [258] and butyrate-producing bacteria were identified in the human esophagus in EoE [253]. However, reports on altered gut microbiota associated with eosinophilic esophagitis are still rare [259]. Thus far, little is known about the impact of SCFAs in eosinophilic esophagitis patients, but a recent investigation revealed that the short chain fatty acid receptor FFA3 is highly expressed in T_H_2 cells in EoE. Further, the authors showed that SCFAs induced the expression of IL-5. In addition, IL-4 stimulation increased the expression of FFA3 in T cells suggesting that a positive feedback loop involving this pathway may contribute to the development or persistence of T_H_2 inflammation in EoE [139,260]. Thus, these findings add controversy to the initial viewpoint that SCFAs have anti-inflammatory effects in eosinophilic diseases.

## 5. Conclusions

Besides their beneficial roles in host defense and tissue homeostasis, activated eosinophils are substantially involved in the pathogenesis of allergic and other inflammatory diseases. Thus, there is still a need for a novel treatment option that specifically targets activated eosinophils. Since apoA-I and apoA-IV have been shown to (i) suppress eosinophil effector function and (ii) eosinophilic diseases such as allergic rhinitis and asthma are linked to apolipoprotein dysregulation, these endogenous proteins or stable peptides derived from them may represent a promising target for the prevention and therapy of eosinophilic inflammation.

It is well known that a Western lifestyle associated with the consumption of calorically rich processed food leads to mostly preventable diseases linked to chronic inflammation. Convincing evidence from preclinical and clinical studies highlights the beneficial effects of microbiota manipulation, high-fiber diet, or directly applied SCFAs on eosinophilic diseases. Moreover, the inhibitory effects of SCFAs such as butyrate on eosinophil effector function and survival has been proven in vitro. Thus, supplementation with specific probiotics to increase SCFA formation or directly applied SCFAs (or specific SCFA receptor agonists) may provide new avenues to manage eosinophilic diseases.

However, future studies are needed to better understand how apolipoproteins and SCFAs modulate eosinophil pro-inflammatory function in vivo, and to assess how the beneficial homeostatic properties of eosinophils are affected.

## Data Availability

Not applicable.

## Abbreviations

ABCA1: ATP-binding cassette subfamily A, member 1; apo: apolipoprotein; BAL: bronchoalveolar lavage; CCAAT: cytosine-cytosine-adenosine-adenosine-thymidine; CCL: chemokine ligand; C/EBP: CCAAT/enhancer binding protein; cysLT: cysteinyl-leukotriene; DNCB: 2,4-dinitrochlorobenzene; DSS: dextran sulfate sodium; EoE: eosinophilic esophagitis; FBA: N-(1-carbamoyl-2-phenylethyl) butyramide; FFA: free fatty acid receptor; FOG: friend of GATA; GATA1: GATA binding protein 1; GM-CSF: granulocyte/macrophage colony stimulating factor; GPR: G-protein-coupled receptor; HDAC: histone deacetylase; HDL: high density lipoprotein; Ig: immunoglobulin; IL: interleukin; ILC2: type 2 innate lymphoid cells; LT: leukotriene; MPO: myeloperoxidase; NF-κB: nuclear factor kappa B; OVA: ovalbumin; PAF: platelet activating factor; RAG2: recombination activating gene 2 protein; RANTES: regulated on activation, normal T cell expressed and secreted; SCFA: short chain fatty acid; SR-B1: scavenger receptor class B, type 1; T_H_2: t helper type 2 cells; TNBS: 2,4,6-Trinitrobenzene sulfonic acid.
